# Ras GAP-related and C-terminal domain-dependent localization and tumorigenic activities of IQGAP1 in melanoma cells

**DOI:** 10.1371/journal.pone.0189589

**Published:** 2017-12-14

**Authors:** Michael Reimer, Elisabeth Denby, Silviya P. Zustiak, Joseph M. Schober

**Affiliations:** 1 Department of Pharmaceutical Sciences, Southern Illinois University Edwardsville, Edwardsville, Illinois, United States of America; 2 Department of Biomedical Engineering, Saint Louis University, Saint Louis, Missouri, United States of America; University of Illinois at Chicago, UNITED STATES

## Abstract

IQGAP1 interacts with a number of binding partners through a calponin homology domain (CHD), a WW motif, IQ repeats, a Ras GAP-related domain (GRD), and a conserved C-terminal (CT) domain. Among various biological and cellular functions, IQGAP1 is known to play a role in actin cytoskeleton dynamics during membrane ruffling and lamellipodium protrusion. In addition, phosphorylation near the CT domain is thought to control IQGAP1 activity through regulation of intramolecular interaction. In a previous study, we discovered that IQGAP1 preferentially localizes to retracting areas in B16F10 mouse melanoma cells, not areas of membrane ruffling and lamellipodium protrusion. Nothing is known of the domains needed for retraction localization and very little is known of IQGAP1 function in the actin cytoskeleton of melanoma cells. Thus, we examined localization of IQGAP1 mutants to retracting areas, and characterized knock down phenotypes on tissue culture plastic and physiologic-stiffness hydrogels. Localization of IQGAP1 mutants (S1441E/S1443D, S1441A/S1443A, ΔCHD, ΔGRD or ΔCT) to retracting and protruding cell edges were measured. In retracting areas there was a decrease in S1441A/S1443A, ΔGRD and ΔCT localization, a minor decrease in ΔCHD localization, and normal localization of the S1441E/S1443D mutant. In areas of cell protrusion just behind the lamellipodium leading edge, we surprisingly observed both ΔGRD and ΔCT localization, and increased number of microtubules. IQGAP1 knock down caused loss of cell polarity on laminin-coated glass, decreased proliferation on tissue culture polystyrene, and abnormal spheroid growth on laminin-coated hydrogels. We propose that the GRD and CT domains regulate IQGAP1 localization to retracting actin networks to promote a tumorigenic role in melanoma cells.

## Introduction

Human IQGAP1 was initially characterized as a 190kD protein with ras-GAP homology and calmodulin-binding motifs [1]. Since the initial discovery, many binding partners and indirect interactions with the CHD domain, a WW motif, IQ repeats, ras-GTPase-activating related domain and a conserved C-terminus sequence in IQGAP1 have been identified, which are in turn proposed to mediate a multitude of cellular, health and disease functions [2,3]. Among the many functions, IQGAP1 is known to localize to the leading edge of lamellipodia in multiple cells types where it participates in regulation actin dynamics. IQGAP1 localizes to and in some cases interacts directly with other proteins in the actin leading edge including protein 4.1R [4], N-Wasp, Arp3 [5,6], APC, Rac1, Cdc42 [7], Clasp2 [8], WAVE2 [9] and phosphatidylinositol 4,5 bisphosphate signaling [10]. IQGAP1 is phosphorylated by protein kinase C (PKC) [11], an event that is involved in epidermal growth factor receptor activation [12], and phosphorylation on IQGAP1 serines 1441 and 1443 are known to regulate neurite growth in neuroblastoma cells [13].

In our previous studies we found localization of IQGAP1 in retracting edges in some cells [14], distinctly separated from Arp3 and WAVE2, two markers of active protrusion [15]. IQGAP1 localizes to areas of retraction in B16F1 [14,16] and B16F10 [14] mouse melanoma cell lines, and among the Wnt-receptor-actin-myosin-polarity (WRAMP) complex in the WM239A human melanoma cell line [17]. Although IQGAP1 is proposed to have various functions in progression of cancers [18], oncogenic potential in canine melanoma [19], and chemotherapeutic drug resistance in human melanoma patients [20], nothing is known of the domains needed for cell retraction localization and little is known of IQGAP1 function in the melanoma cell cytoskeleton. Here we examine localization of IQGAP1 deletion mutants to retraction versus protruding cell areas and describe protein knock down phenotypes in B16F10 mouse melanoma cells. Mutants where either the GRD or CT domain was deleted caused a dramatic change in intracellular localization. Instead of normal localization in retracting cell areas, the GRD and CT deletion mutants appeared at the leading edge of lamellipodia. Protein knock down disrupted cell polarity, and growth on both tissue culture polystyrene (TCP) and polyacrylamide (PA) hydrogels in physiologic stiffness range. Our studies demonstrate that IQGAP1 has tumorigenic properties in melanoma and show that intracellular localization, likely as part of the WRAMP complex, is dependent on GRD and CT domains.

## Materials and methods

### Materials

Dulbecco's Modified Eagle's Medium (DMEM, with 4.5 g/L glucose, L-glutamine and sodium pyruvate), 18mm x 18mm #2 glass coverslips, phosphate-buffered saline (PBS, without calcium and magnesium) and 0.05% Trypsin/0.53mM ethylenediaminetetraacetic acid (EDTA) solution were purchased from Corning Life Sciences (Manassas, VA). Mouse laminin isolated from Engelbreth-Holm-Swarm sarcoma, Alexa 647 anti-rabbit antibody, TRITC anti-mouse antibody, Alexa 488 anti-rabbit antibody, Hoechst 33258, Alexa 488 phalloidin, Cy5 anti-rat antibody and sulfosuccinimidyl 6-(4'-azido-2'-nitrophenylamino) hexanoate (sulfo-SANPAH) were purchased from Thermo Fisher Scientific, Inc. (Waltham, MA). Mouse anti-c-myc (clone 9E10) and rabbit anti-WAVE2 (H-110) were from Santa Cruz Biotechnology (Dallas, TX). Rabbit anti-laminin was from Abcam (Cambridge, MA). Mouse anti-IQGAP1 (clone 24) was from BD Biosciences (San Jose, CA). The rabbit anti-laminin polyclonal antibody and Alexa 488 anti-rabbit antibodies were used for measurement of laminin immobilization to polyacrylamide and glass surfaces. The rat anti-tubulin antibody (clone YL1/2) was from Chemicon International. PlusOne Repel-Silane ES (2% solution of dimethyldichlorosilane dissolved in octamethylcyclooctasilane), PlusOne Bind-Silane (γ-methacryloxypropyltrimethoxysilane) and bovine serum albumin (BSA) fraction V lyophilized powder were purchased from GE Healthcare Life Sciences (Pittsburg, PA). Fetal bovine serum (FBS) used for cell culture media was from Atlanta Biologicals, Inc. (Flowery Branch, GA). The B16F10 mouse melanoma cell line was obtained from American Type Culture Collection (ATCC, Manassas, VA). Triton X-100 was purchased from Sigma-Aldrich (St. Louis, MO), penicillin-streptomycin-amphotericin B solution from MP Biochemicals (Santa Ana, CA), Aqua-Poly/Mount from Polysciences, Inc. (Warrington, PA), 40% para-formaldehyde from Electron Microscopy Sciences (Hatfield, PA) and Fugene 6 from Roche Diagnostics (Indianapolis, IN). Two percent bis-acrylamide solution, 40% acrylamide solution, ammonium persulfate (APS) and N,N,N’,N’-tetramethylethylenediamine (TEMED) were purchased from Bio-Rad Laboratories, Inc. (Hercules, CA). pcDNA3-myc-IQGAP1 (myc-IQGAP1-FL) [21] and pEGFP-IQGAP1 (GFP-IQGAP1-FL) [22] were gifts from David Sacks (Addgene plasmids #30118 and #30112). Delta-CHD, delta-GRD, delta-CT IQGAP1 [23], and S1441E S1441D mutants were generously provided by Dr. Alfredo Caceres, Córdoba, Argentina. The S1441A S1443A mutant was made from full length wild type by Mutagenex Inc. (Suwanee, GA). Lentiviral transduction particles for knock down of IQGAP1 (clone TRCN0000428346, target sequence AGGAAACCCTACGGTTATTAA) and pLKO.1-puro control transduction particles were purchased from Sigma Aldrich (St. Louis, MO).

### Preparation of laminin-coated polyacrylamide and laminin-coated glass surfaces

The Young’s modulus of PA gels was controlled through varying the ratios of acrylamide to bis-acrylamide. Stock solutions (**Table 1**) were made and stored at 4°C until use.

**Table 1:** Polyacrylamide hydrogels composition and Young’s modulus.

Elastic modulus (mean kPa +/- 1 s.d.)	40% acrylamide solution (mL)	2% bis-acrylamide solution (mL)	De-ionized water (mL)
1.06 ± 0.27	3.1	0.3	21.6
10.60 ± 3.10	5.0	1.3	18.8
102.52 ± 28.79	7.5	3.1	14.4

For gel preparation, a large hydrophobic surface was prepared by covering a glass plate with a thin layer of Repel-Silane solution and incubating for 15 min at room temperature. Excess Repel-Silane was removed and the glass was polished with a dry Kimwipe. Working Bind-Silane solution was prepared by mixing 8 mL ethanol, 200 μL glacial acetic acid, 30 μL stock Bind-Silane, and 18 mL water. Eighty μL working Bind-Silane solution was added to the top surface of 18mm X 18mm coverslips and incubated for 15 min at room temperature. Excess solution was removed, and coverslips were dried and polished using a Kimwipe. Note that Bind-Silane facilitates PA hydrogel adhesion to the glass coverslips. For preparation of 1 kPa PA hydrogels, coverslips were briefly dipped once in water and dried with a Kimwipe. To initiate polymerization, 5 μL of 10% w/v APS and 0.5 μL TEMED were mixed with 1 mL of the acrylamide-bis-acrylamide stock solutions. Eighty μL of the hydrogel precursor solution was immediately added onto the hydrophobic glass surface and a glass coverslip, Bind-Silane-treated side down, was placed on top of the solution and incubated at room temperature for 1 h. The coverslips with attached hydrogels were carefully peeled from the glass plates and placed in water for 30 min to remove unreacted monomers and initiators. A working concentration of 500 μM sulfo-SANPAH was prepared by diluting a 100 mM stock solution (solubilized in DMSO) with de-ionized (DI) water. Working sulfo-SANPAH solution (350 μL) was immediately added onto the PA hydrogels and exposed to high intensity UV light (254 nm) for 5 min (CL-1000, UVP, Upland, CA). Excess sulfo-SANPAH was removed and hydrogels were washed in 0.3 M PBS, pH = 7.5 for 5 min. A 5 μg/mL working laminin solution was prepared by dilution with 0.3 M PBS, pH = 7.5. Working laminin solution (350 μL) was added to each sulfo-SANPAH-activated hydrogel and incubated at room temperature for 1 h. For laminin absorption onto untreated glass surfaces, coverslips were place on top of 80 μL working laminin solution for 1 h at room temperature. The laminin solution was removed, coverslips (with and without hydrogels) were dipped once in PBS and then placed in 35 mm dishes containing 2 mL growth media prepared with freshly thawed FBS.

### Rheology

For rheology measurements, PA hydrogels were prepared in a slab geometry of 20 mm diameter and 2 mm height. Gel stiffness was measured by rheology (AR 2000ex rheometer, TA Instruments, New Castle, Delaware) with a 20 mm upper parallel plate geometry, oscillatory frequency sweep test of 1–10 Hz, and a 2% constant strain. Young’s modulus was related to the storage modulus by the following equation:

$$E=G^{\prime }2(1+v)$$ (1)

where *v* is the Poisson’s ratio (0.5 for PA gels).

### IQGAP1 mutant localization and microtubule distribution

B16F10 mouse melanoma cells were maintained in growth media (DMEM with penicillin, streptomycin, amphotericin B and 10% FBS) in 25 cm^2^ flasks until 80% confluent. The cells were removed by 2 min incubation with trypsin/EDTA solution and immediately added to the 35 mm dishes containing media at a density of 30,000 cells/cm^2^. For co-transfection, 1 μg myc-IQGAP1-FL or myc-IQGAP1 mutant and 0.2 μg GFP-IQGAP1 was added to 100 μL sterile DMEM and then mixed with 3.5 μL Fugene 6. The transfection mixture was incubated for 20 min at room temperature and then added to the cells in the 35 mm dishes. After incubation for 20 h at 37°C in a 5% CO_2_ incubator, the cells were removed using the trypsin/EDTA solution and then added to dishes containing laminin-coated glass coverslips in 2 mL growth media prepared with freshly-thawed FBS. Dishes were then incubated for 45 min in a 37°C, 5% CO_2_ incubator. Coverslips were removed and fixed for 90 min at 22°C in cytoskeleton-stabilizing buffer (80 mM PIPES, 2 mM EGTA, 3 mM MgCl_2_, pH = 6.9) with 4% paraformaldehyde and 0.1% Triton-X 100. After fixation, coverslips were washed in DI water and blocked for 20 min at 22°C in 2% w/v BSA solubilized in PBS. Coverslips were incubated at 37°C with primary mouse anti-c-myc and rabbit anti-WAVE2 antibodies, washed, and incubated with secondary anti-mouse TRITC and anti-rabbit Alexa 647 antibodies. Coverslips were washed and then mounted onto glass slides using Aqua-Poly/Mount. All images were acquired with a Leica DMIRE2 HC inverted epifluorescence microscope fitted with a 12-bit grayscale CCD camera using a 63X oil immersion lens. Image analysis was performed using Metamorph software. The outline of the cell was traced to determine whole cell fluorescence intensity. For each cell, linescan fluorescence intensity analysis was performed at 3 areas perpendicular to protruding cell edges (defined by WAVE2-positive staining) and 3 areas perpendicular to retracting cell edges (defined by GFP-IQGAP1-FL-positive localization). The length of each linescan was 4 μm. Whole cell and linescan fluorescence intensity measurements were performed on GFP-IQGAP1-FL, myc-IQGAP1-FL, and each of the myc-IQGAP1 mutants. From the whole cell and linescan measurements retraction localization and protrusion localization were calculated as:

$$Retraction\;localization=\left(\frac{CELLwt}{CELLmut}\right)/\left(\frac{Rwt}{Rmut}\right)$$ (2)

$$Protrusion\;loalization=\left(\frac{CELLwt}{CELLmut}\right)/\left(\frac{Pwt}{Pmut}\right)$$ (3)

Where CELL is a whole cell measurement, R is a linescan measurement in a retracting cell edge, P is a linescan measurement in a protruding cell edge, wt is GFP-IQGAP1-FL integrated fluorescence intensity, and mut is myc-tagged IQGAP1 mutant or myc-tagged IQGAP1-FL integrated fluorescence intensity. For the microtubule localization experiments, cells were fixed with -20°C methanol for 5 min then fixed in cytoskeleton buffer, stained and imaged as described above except anti-rabbit Alexa 488 and anti-rat Cy5 secondary antibodies were used. In Metamorph software, lamellipodia outlines were traced and microtubule tips were counted within 2.1 microns from the cell edge for each cell. Data was expressed as the number of microtubules in an area 2.1 X 80 microns, where 80 microns was the average edge length of lamellipodia.

### Spreading, actin cytoskeleton and proliferation in IQGAP1 knock down cells

Knock down studies were performed on B16F10 cells (F10) infected with the lentiviral transduction particles in accordance with the vendor protocol. Lentiviral particles were incubated with a flask of 80% confluent cells for 24 h in the presence of 8 μg/mL polybrene. IQGAP1 knock down (F10 KD) and virus control (F10 VC) cells were selected with puromycin for 3 wks. For spreading and actin cytoskeleton studies, cells were added to dishes with laminin-coated glass coverslips and incubated at 37°C in 5% CO_2_ for 45 min. Samples were fixed for 90 min at 22°C in cytoskeleton-stabilizing buffer containing 4% paraformaldehyde and 0.1% Triton-X 100, washed in DI water, and blocked for 20 min at 22°C in 2% w/v BSA solubilized in PBS. Samples were incubated with the anti-IQGAP1 antibody, washed and then incubated with TRITC anti-mouse antibody, Alexa 488 phalloidin and Hoechst 33258. Coverslips were washed and then mounted onto glass slides using Aqua-Poly/Mount. Images were acquired with a Leica DMIRE2 HC inverted epifluorescence microscope fitted with a 12-bit grayscale CCD camera using a 63X oil immersion lens and analysis was performed using Metamorph software. Actin images were used to define the cell perimeter for whole cell IQGAP1 fluorescence intensity, cell area and cell length measurements. In the rescue experiments, the knock down cells were transfected with human IQGAP1 tagged with GFP (GFP-IQGAP1-FL, Addgene plasmid #30112) 48 h before each assay. The mouse IQGAP1 RNA interference target sequence (AGGAAACCCTACGGTTATTAA) mis-matches the human IQGAP1 sequence (NM_016721.2) by one nucleotide. Cell length was defined as the longest chord through the cell area. For cell proliferation experiments, 12-well plates were seeded at a starting density of 750 cells/cm^2^. At each time point cells were harvested using the trypsin/EDTA solution and suspended in complete growth medium. Samples were analyzed using an Accuri C6 flow cytometer (San Jose, CA). Cell population was gated in the side versus forward scatter plots for determination of cell count data.

### Spheroid growth assay

F10, F10 VC and F10 KD cells were grown to 80% confluency, removed by 2 min incubation with trypsin/EDTA solution and immediately added to 1, 10 and 100 kPa PA hydrogels crosslinked with laminin at a cell density of 1000/cm^2^. Cells were incubated at 37°C in 5% CO_2_ for 72 h and then fixed for 90 min at 22°C in cytoskeleton-stabilizing buffer containing 4% paraformaldehyde and 0.1% Triton-X 100, washed in DI water, and blocked for 20 min at 22°C in 2% w/v BSA solubilized in PBS. Samples were incubated with Alexa 488 phalloidin and Hoechst 33258, washed, and then mounted onto standard glass slides, hydrogel-side up. A drop of Aqua/Poly mount was added onto the hydrogels and then hydrogels were covered with a plain glass coverslip, so that an optical path through the hydrogels were avoided. Images were acquired with a Leica DMIRE2 HC inverted epifluorescence microscope fitted with a 12-bit grayscale CCD camera using a 40X dry objective. Nuclei in raw images were counted for determination of spheroid and extra-spheroid cell number. Nuclei were counted as spheroid cells if they met the following criteria: nuclei were closely packed surrounded by dense f-actin, cells were rounded, and nuclei were visible through a wide z-distance range. Extra-spheroid cells where defined as nuclei within 109 μm from the spheroid center. The extra-spheroid cells exhibited spreading and were located in z-planes near the PA gel surface. In the rescue experiments, the IQGAP1 knock down cells were transfected with GFP-IQGAP1-FL 18 h before seeding onto the hydrogels. For presentations purposes, the following operations where applied to representative z-series of nuclei and actin image sets: low pass filter, no neighbors deconvolution and maximum projection. All image analysis and z-series operations were performed using Metamorph software.

### Statistical analysis

Experiments were repeated at least 3 times independently on different days. A single condition within an experiment consisted of a coverslip or well. For IQGAP1 mutant localization studies, a minimum of 2 cells per image, from a minimum of 12 images were analyzed for each coverslip. The cell proliferation assay, consisting of 3 replicates, was repeated 3 times and the counts were averaged. Statistical significance between multiple samples was tested by one-way ANOVA followed by a Tukey’s post hoc test, where p<0.05 was considered significant. Results are reported as means ± standard error for all graphs except the proliferation assay where the results were reported as means ± 1 standard deviation. All data analysis was performed in GraphPad Prism.

## Results

### Localization of IQGAP1 mutants to protruding versus retracting cell areas, and microtubule distribution in lamellipodia

**Fig 1:**
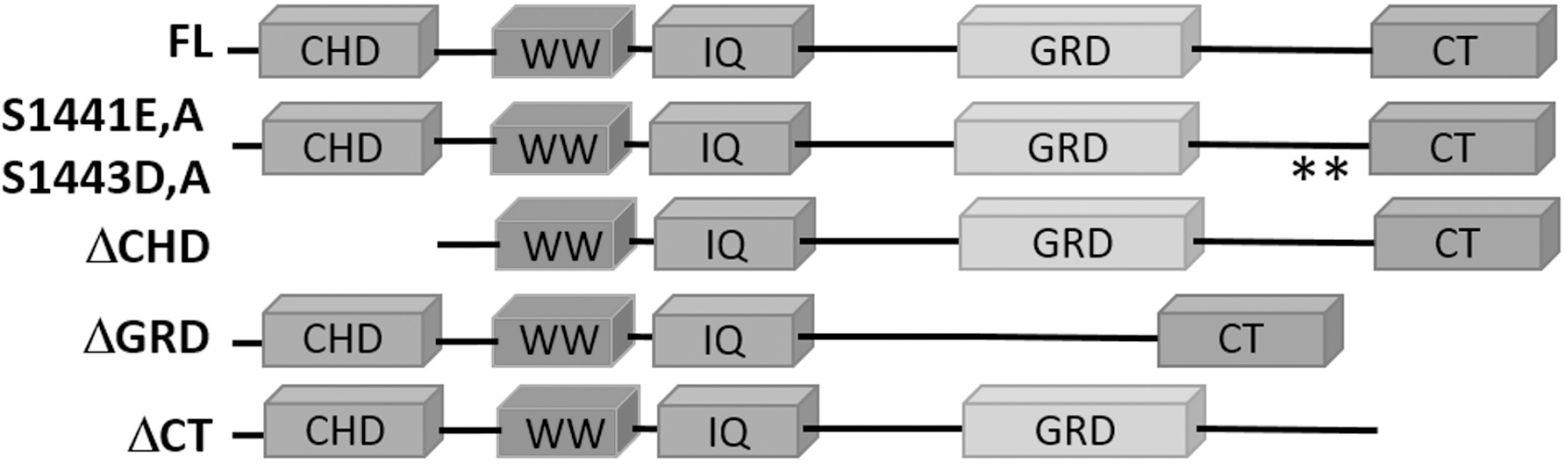
Myc-tagged IQGAP1 mutants used in this study. FL, full length wild type protein; CHD, calponin-homology domain; WW, conserved tryptophan repeats; IQ, isoleucine-glutamine calmodulin-binding motif; GRD, ras-GTPase-activating protein-related domain; CT, a conserved C-terminal domain. The two asterisks highlight positions where serines at positions 1441 and 1443 were substituted with glutamate and aspartate, or alanines.

IQGAP1 has been extensively reported to localize to protruding cell edges through association with other protein effectors in the actin protrusion machinery [4–8,24]. More recently, IQGAP1 was discovered to localize to retracting edges in mouse [14,16] and human melanoma cell lines [17]. We previously reported that the subcellular localization of IQGAP1 was dependent on actin cytoskeleton retraction, and of the cell lines examined, IQGAP1 was most distinctly separated from WAVE2 in B16F10 mouse melanoma cells [14]. Here, we aimed to identify the domains needed for localization of IQGAP1 to retracting edges and further characterize its role in single cell motility and melanoma tumor growth. We developed a mutant localization assay using six myc-tagged IQGAP1 constructs: 3 domain deletions mutants, 2 phosphorylation site mutants, and 1 full length control (**Fig 1**). Phosphorylation by PKC [11] between IQGAP1 GRD and CT domains at S1441 and S1443 regulates the actin cytoskeleton in neuroblastoma cells to promote neurite outgrowth.

**Fig 2:**
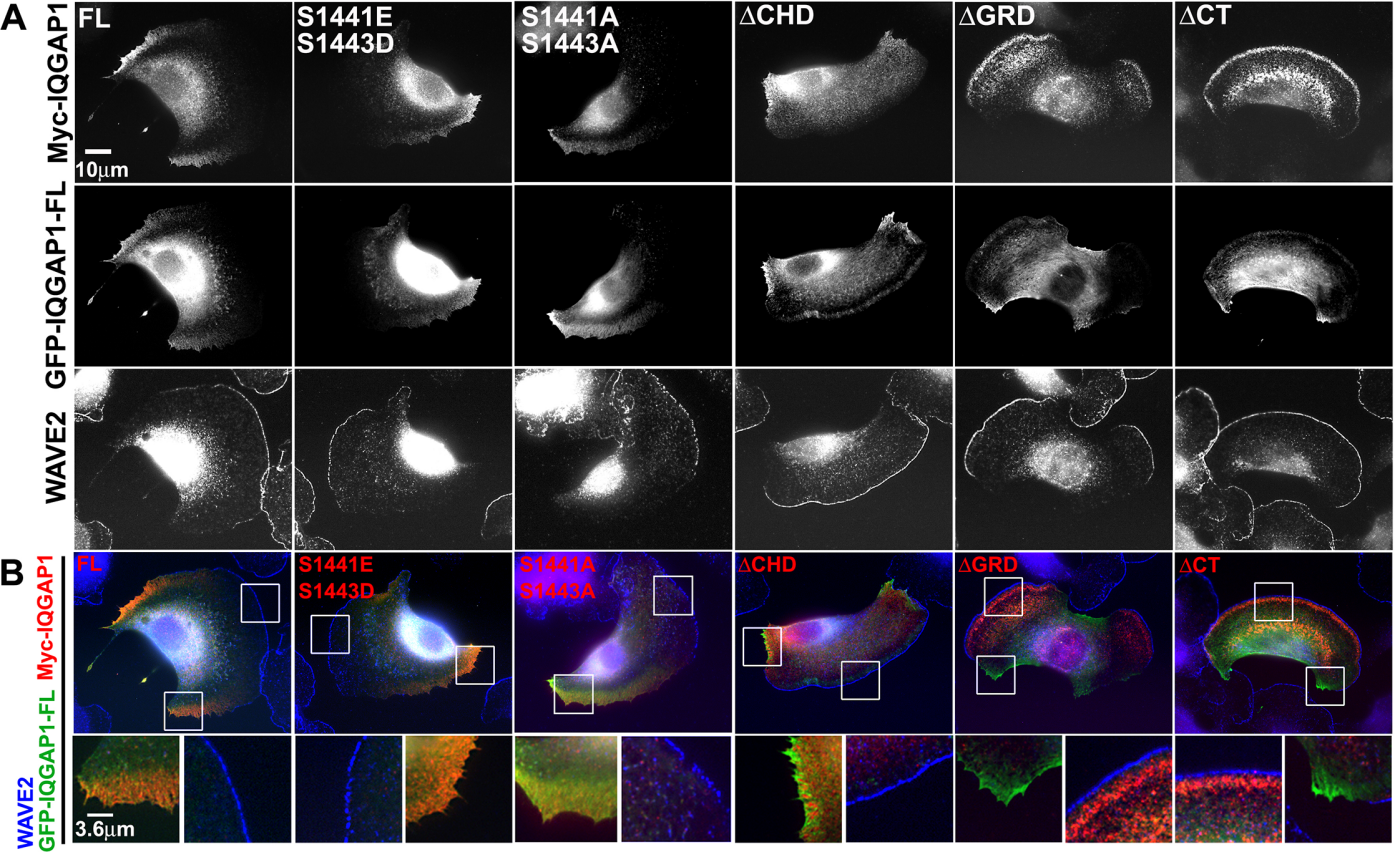
Co-localization of myc-IQGAP1 mutants with WAVE2 and GFP-IQGAP1. B16F10 cells were co-transfected with a myc-tagged IQGAP1 mutant and GFP-IQGAP1-FL, and stained with anti-WAVE2. **A)** Representative images for myc-tagged IQGAP1 full length (FL) and mutant myc-tagged sequences (S1441E S1443D, S1441A S1443A, delta-CHD, delta-GRD and delta-CT) with corresponding GFP-IQGAP1-FL and WAVE2 images. **B)** Color-combine images of myc-tagged mutants (red), GFP-IQGAP1-FL (green) and WAVE2 (blue) with enlarged inset views of GFP-IQGAP1-FL positive and WAVE2 positive areas for each cell.

**Fig 3:**
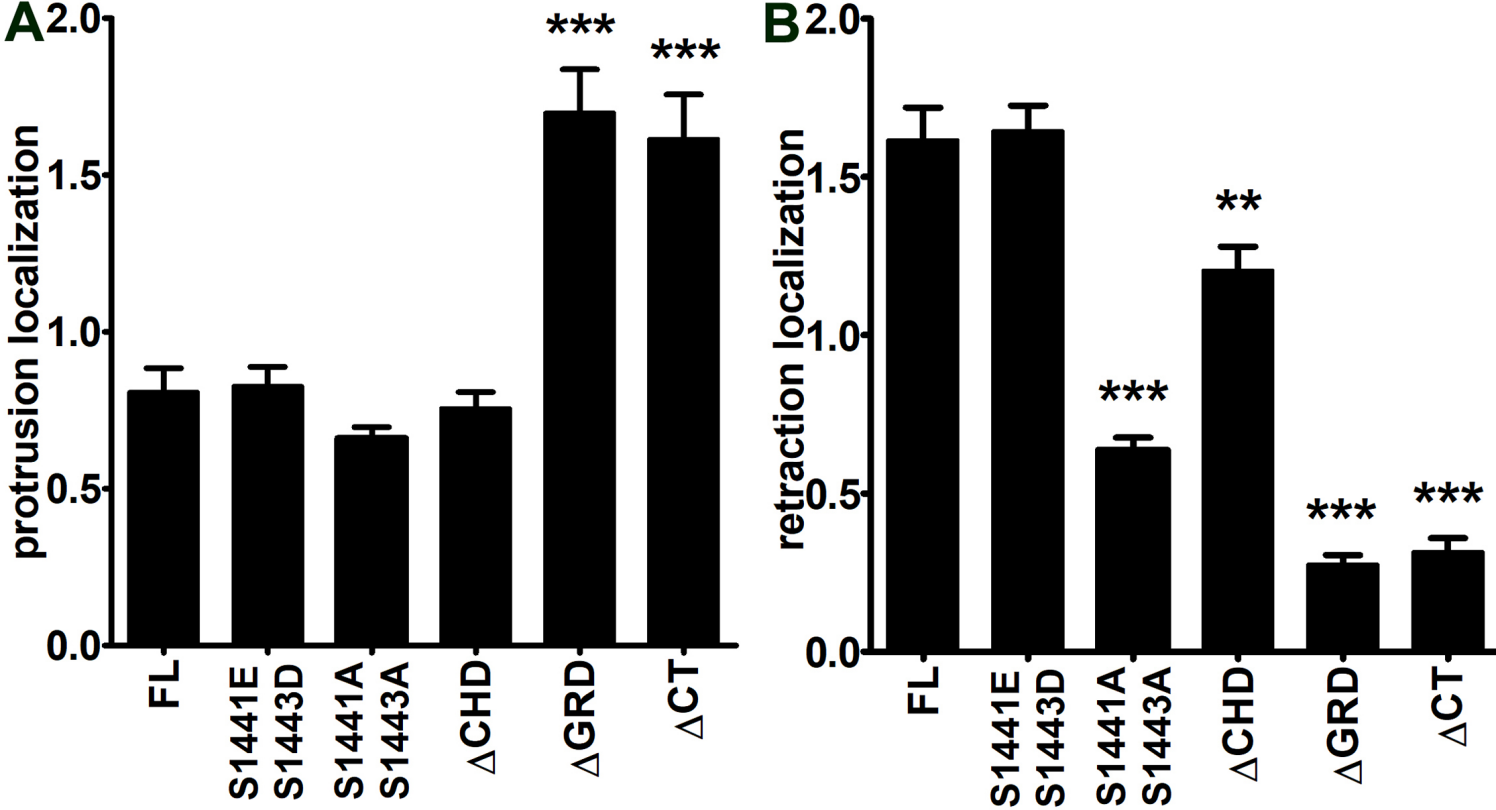
Localization of IQGAP1 mutants to areas of cell retraction versus cell protrusion. Images were analyzed for co-localization of myc-tagged IQGAP1 full length (FL) and mutants (S1441E S1441D, S1441A S1443A, delta-CHD, delta-GRD and delta-CT) with GFP-IQGAP1-FL or WAVE2 as described in Materials and Methods. **A)** Protrusion localization is defined as normalized fluorescence in WAVE2-positive areas. **B)** Retraction localization is defined as normalized fluorescence in GFP-IQGAP1-FL-positive areas. **p<0.01, ***p<0.001 Tukey’s post hoc test compared to FL. n = 30–33 cells for each condition. Bars = mean +/- s.e.m.

**Fig 4:**
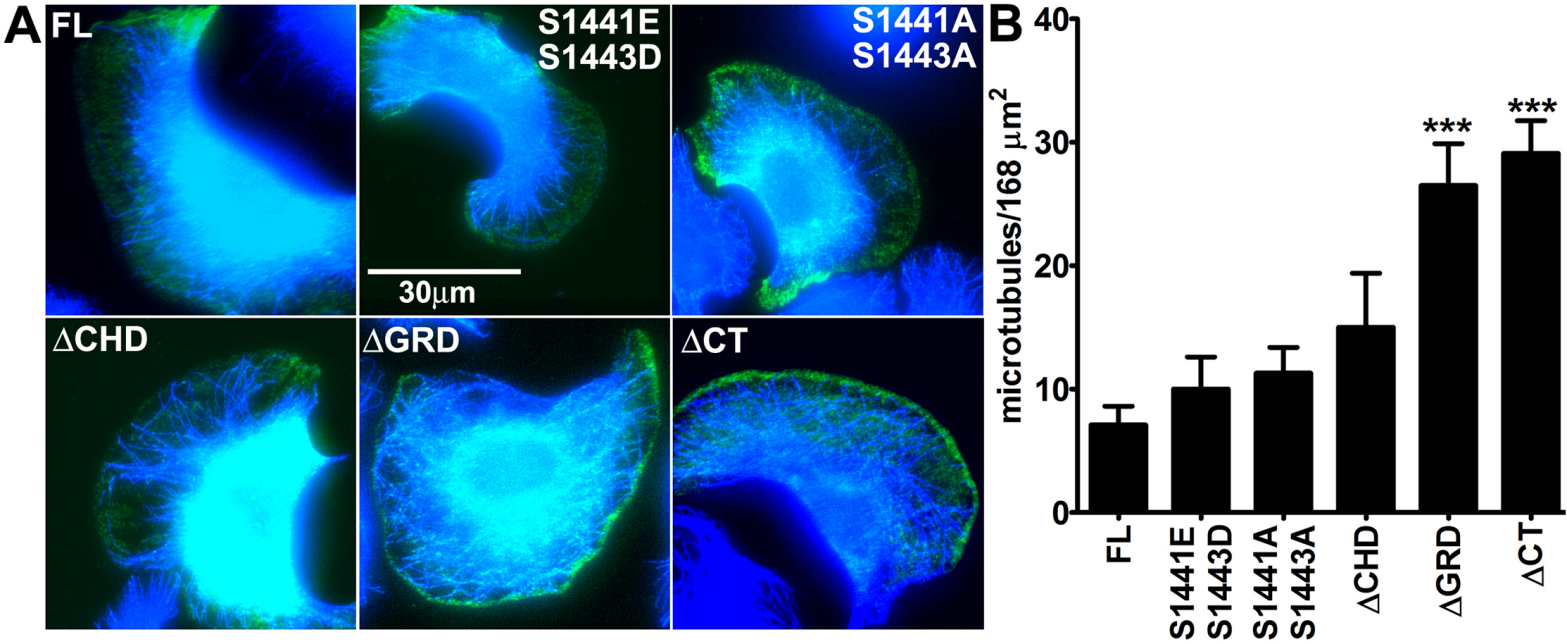
Distribution of microtubules in lamellipodia in cells expressing IQGAP1 mutants. A) Representative color combine images of IQGAP1 full length (FL) or IQGAP1 mutants (S1441E S1441D, S1441A S1443A, delta-CHD, delta-GRD and delta-CT) in green and microtubules in blue. B) Average number of microtubule ends within 2.1μm of the lamellipodium edge. The average edge length of lamellipodia was 80μm. ***p<0.001 Tukey’s post hoc test compared to FL. n = 11–15 cells for each bar. Bars = mean +/- s.e.m.

We focused localization studies on the population of cells with a polarized shape forming broad lamellipodia and edges of retraction, a typical morphology observed in B16 melanoma cells on two dimensional (2D) environments [25,26]. We adopted a broad definition of retraction to include any edge that is moving toward the cell center. This definition includes areas outside the cell tail, and may undergo cycles of actin protrusion and retraction [27–29]. Subcellular measurements in B16F10 cells were made in areas of active protrusion, marked by WAVE2 localization, and in areas of cell edge retraction, marked by full length GFP-IQGAP1 localization [26]. The panels in **Fig 2** show representative images for each myc-tagged construct, GFP-IQGAP1-FL, WAVE2, color-combined whole cell images, and color-combined enlarged insets in areas of protrusion versus retraction. We observed WAVE2 localization at the leading edge of lamellipodia and GFP-IQGAP1 in areas of cell retraction irrespective of mutant IQGAP1 expression, indicating that the mutant expression did not disrupt the overall polarized shape (**Fig 2A**). A notable observation was a dramatic change in localization of the ΔGRD and ΔCT mutants away from retracting edges to protruding edges. Both the ΔGRD and ΔCT mutants localized to protruding lamellipodia behind WAVE2, about 1 μm from the cell edge (**Fig 2B**). Among the mutants tested, ΔGRD and ΔCT had the largest decrease in retraction localization, and were the only mutants that had elevated localization to protruding edges (**Fig 3A**). Retraction localization of the S1441A S1443A and ΔCHD mutants was decreased to a lesser extent, while protrusion localization was equal to full length (**Fig 3B**). We examined the distribution of microtubules in lamellipodia in cells expressing myc-IQGAP1 full length or each of the mutants (**Fig 4A**). The phosphorylation site and ΔCHD mutations did not alter microtubule distribution compared to cells expressing full length IQGAP1 which had 7.1 +/- 1.5 microtubules within 2.1μm from the lamellipodium edge. In contrast, we observed increased number of microtubules in cells expressing either the ΔGRD or ΔCT mutant (**Fig 4B**).

### Cell spreading and actin cytoskeleton in IQGAP1 knock down cells

**Fig 5:**
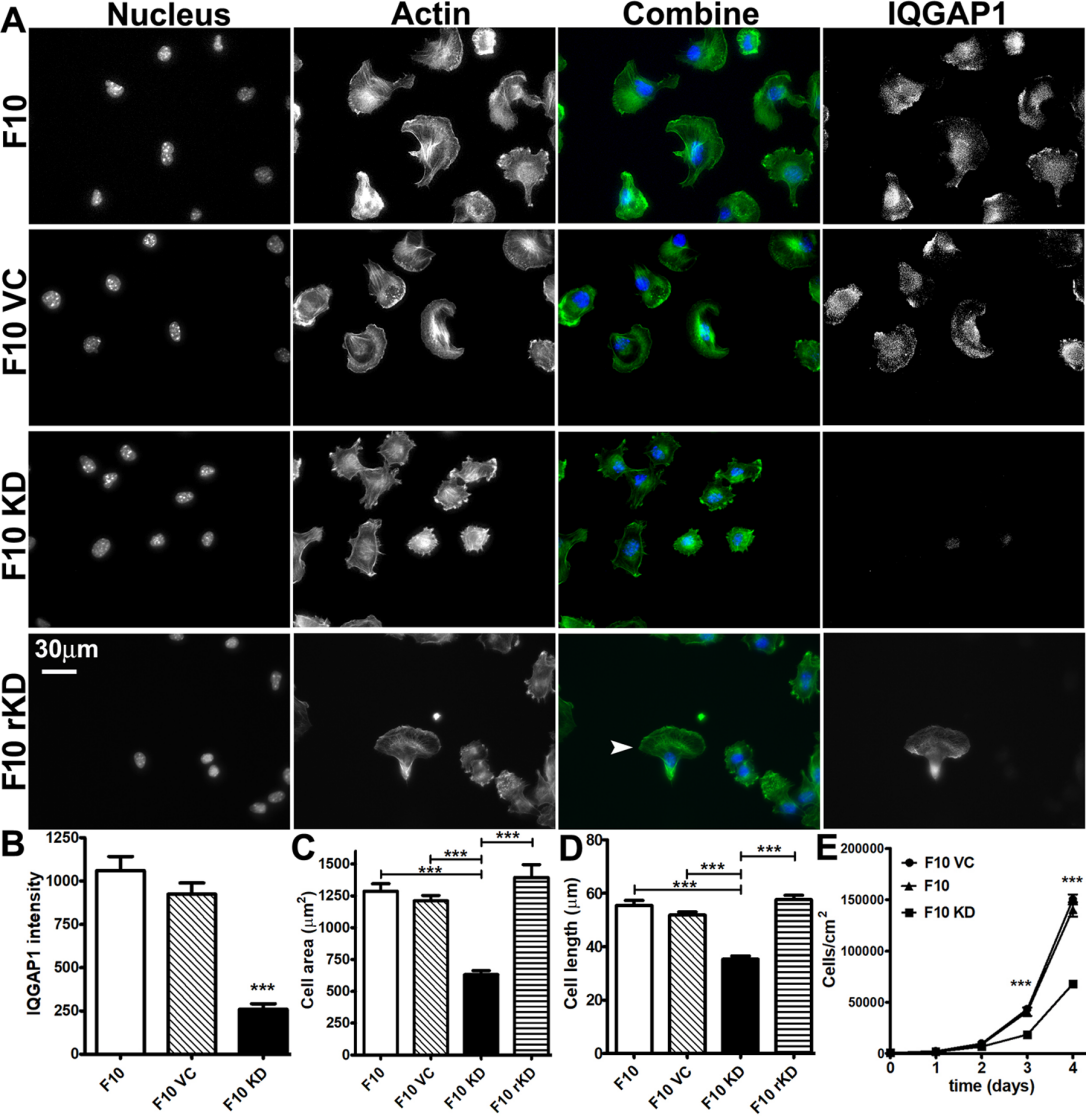
Effect of IQGAP1 knock down on cell morphology and cell division. Native B16F10 mouse melanoma cells (F10), virus control cells (F10 VC), IQGAP1 knock down cells (F10 KD) and F10 KD cells transfected with GFP-IQGAP1-FL (F10 rKD) were incubated on laminin-coated glass coverslips for 45 min (A-D) or in 12 well TCP plates for 4 d (E). (A) Representative images show individual nucleus, actin, IQGAP1 channels with combined nucleus (blue) and actin (green) images. The arrowhead marks a cell expressing GFP-IQGAP1. B) IQGAP1 protein quantified through immunofluorescence intensity. n = 38–40 cells for each condition. C) Projected cell area from actin images. n = 38–44 cells for each condition. D) Cell length expressed as the longest chord through the cell measured from actin images. n = 38–44 cells for each condition. E) Number of cells per cm^2^ at 0, 1, 2, 3 and 4 d. n = 9 wells over 3 experiments for each time point. ***p<0.001 Tukey’s post hoc test as indicated by the brackets in C and D, or comparing F10 KD to F10 and F10 VC in B and E. Bars = mean +/- s.e.m. Time points = mean +/- 1 s.d.

To date, only a few reports describe IQGAP1 localization to areas of cell retraction [14,16,17]. Knock down studies have been performed on cells where IQGAP1 normally localizes to protruding edges with Arp3 [5] or cell tight junctions [30]. Here, stable knock down of IQGAP1 in B16F10 cells was associated with decreased formation of lamellipodia after 45 min on laminin-coated glass coverslips (**Fig 5A**). A 75% decrease in IQGAP1 immunofluorescence (**Fig 5B**) was associated with a 48% and 33% decrease in cell spreading area and cell length, respectively (**Fig 5C** and **5D**). The effects of protein knockdown on lamellipodia formation, area and spreading were reversed in cells transfected with GFP-IQGAP1-FL (F10 rKD in **Fig 5A**, **5C** and **5D**). Thus, although IQGAP1 localized almost exclusively to retracting areas in this cell type, protein depletion interfered with lamellipodia formation and cell spreading in response to laminin.

### Growth of IQGAP1 knock down cells on TCP and PA-hydrogels

**Fig 6:**
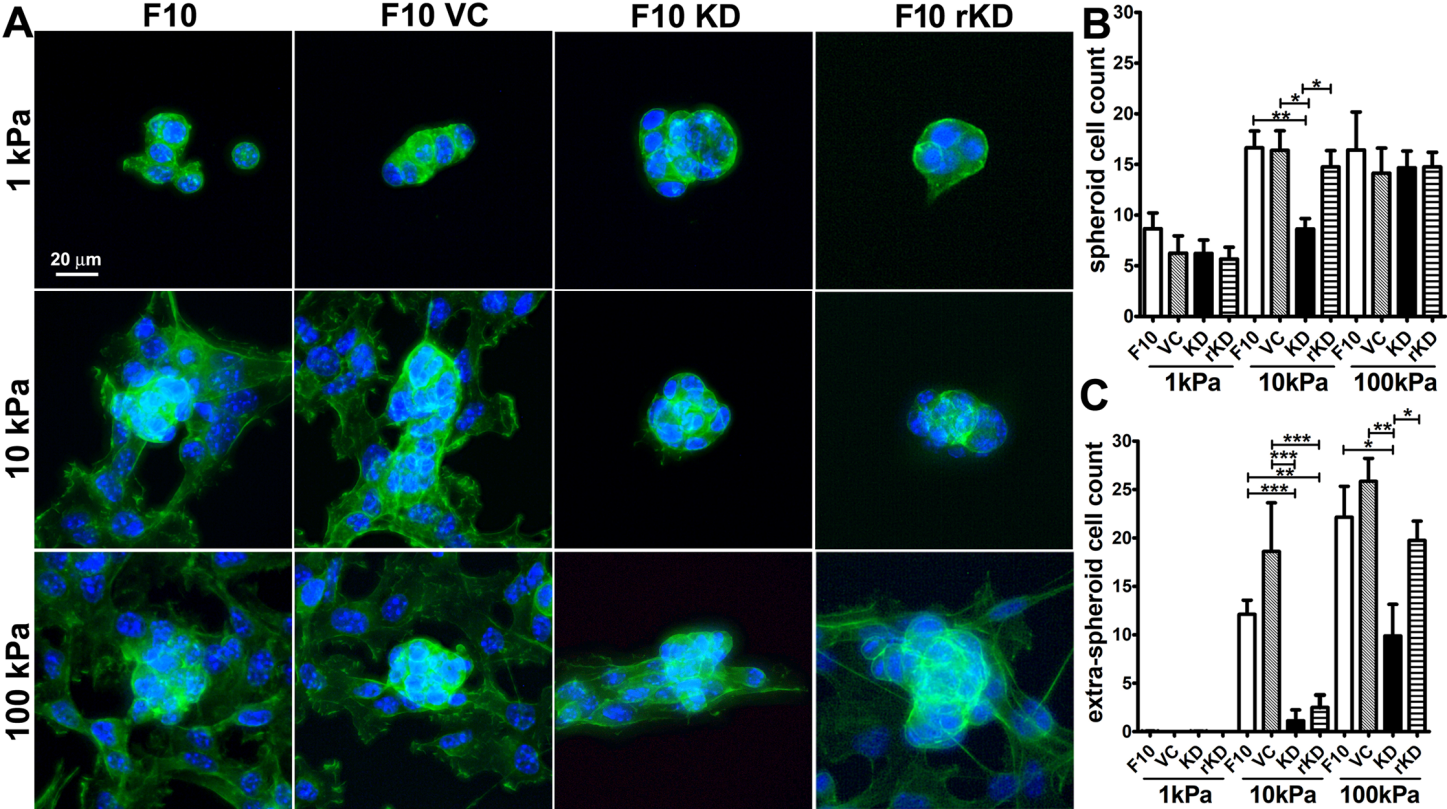
Growth of B16F10 (F10), virus control (F10 VC), IQGAP1 knockdown (F10 KD) and F10 KD cells expressing GFP-IQGAP1-FL (F10 rKD) on laminin-coated PA hydrogels. Cells were added at low density and incubated for 4 d on 2D polyacrylamide hydrogels of 1, 10, or 100 kPa Young’s modulus coated with laminin. After incubation, samples were fixed and stained for DNA and actin. (A) Representative images for each condition show combined DNA (blue) and actin (green) channels. (B) Spheroid and (C) extra-spheroid growth of cells on laminin-coated PA hydrogels. The number of cell nuclei were counted inside the spheroid and in the area outside the spheroid within a 109 μm radius from the spheroid center (extra-spheroid). *p<0.05, **p<0.01, ***p<0.001 Tukey’s post hoc test. Bars = mean +/- s.e.m. n = 5–30 spheroids for each condition.

A number of studies link IQGAP1 to cancer through roles in tissue invasion, alterations in cell-cell junctions, angiogenesis, cell proliferation [18,31] and more recently, resistance to a BRAF inhibitor used to treat melanoma [20]. We examined the role of IQGAP1 on cell growth on laminin immobilized onto tissue culture polystyrene (TCP) and PA hydrogel surfaces. Cells suspended in growth media were added in 12-well TCP plates at a starting density of 1500 cells/cm^2^ and proliferation was monitored for 3 d (**Fig 5E**). On TCP during the rapid growth phase at days 1 and 2, knock down of IQGAP1 did not alter cell density; however, at day 3 F10 KD cell density was decreased 51% (**Fig 5E**). Thus, similar to other non-cancer and cancer cell types [32–34], knockdown of IQGAP1 in mouse melanoma cells decreased cell proliferation. We next evaluated growth of knock down cells on laminin immobilized onto 2D PA hydrogels of 1, 10 and 100 kPa Young’s modulus, a range overlapping with physiologic stiffness of skin [35,36]. After 4 d of growth, we observed two main cell morphology types in the actin images: cells were either part of a spheroid or in a single layer surrounding a spheroid (**Fig 6A**). The amounts of spheroid versus extra-spheroid cells were dependent on PA hydrogel stiffness and the presence of IQGAP1. On the 1 kPa hydrogels, cell growth was restricted to spheroid morphology and on 10 and 100 kPa gels, cell growth morphology was mixed. While IQGAP1 knock down decreased spheroid cell count only on 10 kPa hydrogels (**Fig 6B**), the most dramatic effect of IQGAP1 knock down was on extra-spheroid cell count (**Fig 6C**). IQGAP1 knock down decreased extra-spheroid cell count by 90% on 10 kPa hydrogels and 55% on 100 kPa hydrogels compared to F10 control cells. Thus, depletion of IQGAP1 had a more pronounced effect on decreasing the number of cells outside the spheroid rather than the spheroid size itself. Furthermore, the effect of protein knockdown on spheroid count on 10 kPa hydrogels and extra-spheroid count on 100 kPa hydrogels was completely and partially reversed in cells transfected with GFP-IQGAP1-FL (F10 rKD, **Fig 6A–6C**).

## Discussion

IQGAP1 has an established role in regulating actin network assembly at the leading edge during cell migration [2]; however, a few publications report localization to areas of cell retraction. IQGAP1 localization is restricted to retraction in B16F1 [14,16] and B16F10 mouse melanoma cell lines, and part of the WRAMP complex in the WM239A human melanoma cell line [17]. Thus, these cell lines provide a unique opportunity for the study of IQGAP1 in a yet uncharacterized function. Here we examined IQGAP1 mutant localization and knock down effects in B16F10 mouse melanoma cells. Using a method that normalizes the mutant expression to the full length wild type and corrects for varying mutant expression level, we identified domains needed for normal retraction localization. Deletion of the GRD or CT domain caused the largest decrease in localization, while removal of the CHD domain had only a small effect on retraction localization, possibly through loss of interaction between CHD and calmodulin or actin [37]. As we examined localization to areas of cell protrusion, surprisingly, the GRD and CT deletion mutants localized to cell lamellipodia just behind WAVE2 at the leading edge.

In a cell type where normal IQGAP1 localization is found only in areas of retraction, expression of mutants without the GRD or CT domain caused localization to areas of active protrusion in lamellipodia. In most other cells, where IQGAP1 is found near the leading edge in lamellipodia, deletion of the CHD domain is expected to disrupt localization resulting from loss of interaction with f-actin or N-WASP [38]. Notably, we observed protrusion localization only with the GRD and CT deletion mutants indicating a unique mechanism. Our studies also suggested that IQGAP1 phosphorylation state alters localization, but only in areas of cell retraction. Localization of the phosphomimetic mutant was the same as full length wild type while the alanine substitution mutant was lower in areas of B16F10 retraction, suggesting that the baseline phosphorylation is normally very high, which may favor an open IQGAP1 conformation [39]. In neuroblastoma cells, the phosphomimetic mutant increased neurite outgrowth while the alanine substitution had no discernable effect [13], highlighting distinct IQGAP1 regulation in melanoma cells.

In melanoma, IQGAP1 may aberrantly associate in areas of retraction through an interaction with a component of the WRAMP complex [17] initiated by cell adhesion to laminin [40]. In our experimental conditions, the melanoma cells are indeed attached to laminin immobilized on glass or hydrogel surfaces. Laminin-dependent activation of melanoma cell adhesion molecule (MCAM) could lead to IQGAP1 recruitment followed by intracellular calcium increase [17], findings congruent with our previous observations showing myosin IIA and calmodulin increase in retraction areas along with IQGAP1 [14]. The rise in free intracellular Ca2+ may promote binding of calmodulin to IQGAP1 which in turn inhibits IQGAP1 interaction with cortical actin filaments [41]. IQGAP1 could be present in the WRAMP complex in other cell types during retraction, such as HUVEC, C2C12 [17] and HT-1080s human fibrosarcoma cells [42].

Our results raise the question of which binding partner is needed for proper localization to retracting areas in cell types such as melanoma. Reports point to interaction between Rho family small GTPases with IQGAP1 C-terminal domains which could regulate Rho kinase-myosin II dependent retraction. Rho inhibitors disrupt the WRAMP complex in human melanoma cells [17] and IQGAP1 is known to interact with RhoA [43,44]. While promoting activity of RhoA, IQGAP1 may also decrease activity of Rac1 [45] in the cell tail during melanoma migration, which may require interactions with C-terminal domains of IQGAP1. Thus, Rho GTPase interaction with IQGAP1 may explain why the GRD and CT deletion mutants in our studies failed to localize normally to areas of retraction. Although C-terminal dependent IQGAP1 localization as part of the WRAMP complex or association with Rho small GTPases are attractive mechanism for localization to areas of cell retraction, we cannot rule out other interacting partners such as Dia1 [38] or phosphatidylinositol 4,5-bisphosphate [39] with a sequence in the IQGAP1 C-terminal domain. Our studies support a model where the preferred localization in melanoma is through the C-terminus with binding partners in areas of actin retraction; however, when the C-terminus interaction is disrupted, interaction through the CHD domain may re-localize IQGAP1 to actin network protrusion. Interestingly, we found increased number of microtubules in the lamellipodia of cells expressing the GRD or CT deletion mutants. The elevated microtubule distribution near the lamellipodium edge may result from mislocalization of the ΔGRD and ΔCT mutants combined with altered Rac1, mDia1 or phosphatidylinositol phosphate kinase interaction, causing change in retrograde movement of actin or microtubule plus-end growth. Both processes, either decreased retrograde movement of the actin network or increased net growth of microtubule ends, would result in more microtubules near the lamellipodia edge. If Rac1 activity in lamellipodia is dependent on IQGAP1 interaction [7] then GRD and CT deletion mutants may slow retrograde movement of actin, allowing for more microtubules to enter the lamellipodia. If Rac1 activity is increased by some other mechanism, then microtubule growth may also increase [46]. Alternatively, loss of mDia1 interaction with IQGAP1 could decrease proper mDia1 localization [47] resulting in decreased actin network growth [48]. Thus, recruitment of the GRD and CT deletion mutants to the leading edge through phosphatidylinositol phosphate kinase binding may act in a dominant negative fashion to alter lamellipodium growth [10]. These and other potential mechanisms are likely areas of our further investigation.

We performed knock down studies in melanoma cells which have the unusual property where IQGAP1 localizes exclusively to areas of retraction. Through the many interactions and binding partners, IQGAP1 is proposed to underlie progression of several types of cancers [18,31] and more recently, drug resistance in melanoma [20]. Laminin, known to promote cancer progression [49], may enhance tumor cell migration through activation of MCAM [40], IQGAP1 recruitment and subsequently myosin II retraction [17,50]. We observed that IQGAP1 knock down cells on TCP and in the extra-spheroid space on laminin-coated PA hydrogels were decreased to a much greater extent compared to spheroid cell counts. Directional motility is an orchestrated process where a cell must maintain polarity and coordinate retraction of the tail with protrusion of the lamellipodium in a tightly controlled temporal and special manner [51]. Thus, by a distinct mechanism potentially through WRAMP interaction or Rho family GTPases described above, IQGAP1 in melanoma functions to upregulate tissue invasion and metastasis rather than primary tumor growth. In addition, we noted recoveries of knock down cell counts in both spheroid and extra-spheroid spaces on the 100 kPa hydrogels suggesting that phenotypes may be surmounted in higher stiffness tissues [52]. Higher stiffness may activate pathways downstream of IQGAP1 therefore bypassing the need for fully functional IQGAP1 and restoring migration and proliferation [53].

In conclusion, we characterized localization of IQGAP1 deletion mutants and protein knock down phenotypes in mouse melanoma cells. These studies are important because little is known about IQGAP1 function in cell types where localization is restricted to retracting actin networks. Removal of the GRD or CT domains not only decreased localization to areas of cell retraction, but unexpectedly increased localization to actively protruding lamellipodia behind WAVE2 potentially through a low affinity CHD domain interaction. Our studies suggest that IQGAP1, resulting from aberrant C-terminal localization with the WRAMP complex or other pathway in cell tail retraction, plays a tumorigenic role in melanoma.
